# Cognitive-behavioral teletherapy for children and adolescents with mental disorders and their families during the COVID-19 pandemic: a survey on acceptance and satisfaction

**DOI:** 10.1186/s13034-022-00494-7

**Published:** 2022-07-28

**Authors:** Lea Meininger, Julia Adam, Elena von Wirth, Paula Viefhaus, Katrin Woitecki, Daniel Walter, Manfred Döpfner

**Affiliations:** 1 Faculty of Medicine and University Hospital Cologne, School of Child and Adolescent Cognitive Behavior Therapy (AKiP), University of Cologne, Pohligstr. 9, 50969 Cologne, Germany; 2 Department of Child and Adolescent Psychiatry, Psychosomatics and Psychotherapy, Faculty of Medicine and University Hospital Cologne, University of Cologne, Robert-Koch-Str. 10, 50931 Cologne, Germany

**Keywords:** Teletherapy, Video therapy, COVID-19, Acceptance, Treatment satisfaction, Child, Adolescent, Outpatient behavior therapy

## Abstract

**Background:**

The COVID-19 pandemic is challenging for health care systems around the world. Teletherapy (psychotherapy conducted via videoconference) for children and adolescents offers a promising opportunity not only to provide treatment during social distancing restrictions but also to reduce treatment barriers that might prevent families from seeking care independent of the pandemic. Therefore, it is highly important to examine the implementation and especially the acceptance of and satisfaction with teletherapy.

**Methods:**

Therapists of 561 patients and parents of 227 patients (total 643 patients) aged 3–20 years treated at a university outpatient unit rated their experiences with teletherapy.

**Results:**

Following the outbreak of COVID-19, 73% of the patients switched from face-to-face treatment to teletherapy. Both therapists and parents were mainly satisfied with teletherapy and did not report negative impacts on treatment satisfaction or the therapeutic relationship. Stress from COVID-19, age, gender, duration of treatment, psychosocial functioning, and psychopathology were associated with satisfaction, but correlations were low. Sixty-six percent of parents and 53% of therapists intended to use teletherapy in the future.

**Conclusions:**

Teletherapy during the COVID-19 pandemic was well accepted by both parents and therapists. Certain patient characteristics were related to satisfaction.

*Trial registration* The study was retrospectively registered in the German Clinical Trials Register (DRKS00028639).

**Supplementary Information:**

The online version contains supplementary material available at 10.1186/s13034-022-00494-7.

The COVID-19 pandemic has changed the lives of children and adolescents across the world. The first confirmed cases in Germany occurred in January 2020 [44] and lockdown was implemented in March 2020 [7]. Consequently, many outpatient units and private practices for child and adolescent psychiatry and psychotherapy reduced face-to-face treatments to a minimum, and teletherapy (psychotherapy delivered via videoconference) was often the only option to continue outpatient treatment. The German National Association of Statutory Health Insurance Physicians [Kassenärztlichen Bundesvereinigung (KBV)] reacted quickly and abolished several restrictions regarding the delivery of online consultations in routine care [17].

Even before the onset of the COVID-19 pandemic, several studies demonstrated high levels of acceptance of teletherapy and satisfaction with this form of treatment delivery in children and adolescents with mental disorders [21, 31] and their parents [28, 38], with some even preferring teletherapy and telepsychiatry (video-based psychiatric consultations) over face-to-face treatments [16, 32, 36]. For example, Xie et al. [45] found no difference in satisfaction between video treatment and face-to-face treatment in parents of children with ADHD. In a recent survey, children treated at a university outpatient unit and their parents reported moderate to good satisfaction with teletherapy during the COVID-19 pandemic [3].

Mental health providers have also reported being generally satisfied with teletherapy for children and adolescents (e.g. [9, 10]. The level of satisfaction with technology-based, remotely delivered treatment seems to be associated with a number of factors. Myers, Valentine and Melzer [29] evaluated telepsychiatry sessions provided by psychiatrists at a children’s hospital in the USA and found that parents were more satisfied the more frequent the online treatment and that parents of school-aged children were more satisfied than parents of adolescents. In contrast, a qualitative study undertaken during the 2020 lockdown in Australia found that clinicians mainly viewed younger children as disadvantaged by mental health services via telehealth and saw advantages especially for adolescents [22].

Therapists have also expressed concerns about teletherapy and other forms of telehealth. For example, Rees and Stone [37] reported that the therapeutic relationship was rated as significantly lower in video-delivered therapy compared to face-to-face sessions. Clinicians have also endorsed concerns regarding technological difficulties, logistics, and information sharing [10]. Moreover, several studies revealed that previous experience with internet-based forms of therapy influences therapists’ satisfaction with online therapy [20, 39].

Another factor that has been found to be associated with satisfaction with internet-based treatments is the severity of the child’s mental disorder. Internet-based programs were reported to be more accepted for mild to moderate disorders than for severe disorders [20]. Based on these findings, it can be assumed that previous experiences with video-delivered treatments, as well as children’s age and symptom severity, influence the satisfaction with teletherapy.

Despite the sharp increase in the use of teletherapy during the COVID-19 pandemic in Germany ([15, 35], relatively few studies have systematically investigated this form of treatment. Overall, studies evaluating service delivery and outcome variables of telepsychiatry in children and adolescents are rare [2]. Studies examining teletherapy within routine care are mostly case studies or comprise (very) small samples (e.g., [16, 24, 36], or had methodological difficulties. For example, some studies asked mental health providers about their attitudes towards videotherapy, regardless of whether they had practical experience [20, 39], while others mainly used qualitative evaluation methods [18, 19].

During the first wave of the COVID-19 pandemic in 2020, the University Hospital Cologne limited the number of face-to-face outpatient contacts to a minimum, with face-to-face psychotherapy sessions for children and adolescents at the outpatient unit of the School for Child and Adolescent Cognitive Behavior Therapy (AKiP) only offered for acute crises, severe cases, and when teletherapy was not an option. To maintain routine care, teletherapy was immediately implemented at the beginning of the first lockdown. Fortunately, the technical requirements were already installed since an online outpatient unit had been set up in 2019. Moreover, the unit had gained experience with video-delivered treatment for patients with tic disorders and obsessive–compulsive disorders during an earlier trial [1, 43]. However, most of the therapists working at AKiP did not have any experience with teletherapy before the lockdown. With the beginning of the lockdown, they were instructed to transform as many of their treatments as possible into video sessions.

This study aims to evaluate the implementation and especially the acceptance of and satisfaction with teletherapy in a large sample of patients (children and adolescents) of the outpatient unit for cognitive behavioral therapy at AKiP. In contrast to most previous studies, the changeover to teletherapy occurred rapidly and mainly unprepared due to the exceptional pandemic situation. Therapist and parent ratings were assessed to examine (1) the extent to which teletherapy was implemented and accepted within routine outpatient care under pandemic conditions and (2) the satisfaction with teletherapy and potential factors influencing treatment satisfaction.

## Methods

### Participants

Therapists and parents of all patients treated at the outpatient unit of AKiP in the first quarter of 2020 (*N* = 878) were asked to participate in a survey on the impact of the COVID-19 pandemic on the patient’ well-being and treatment. All therapists held a Master’s degree in psychology or education and were in the second half of their training in child and adolescent CBT, which encompasses 5 years. The psychotherapy sessions were guided by an accredited CBT supervisor (one supervision session every fourth therapy session).

### Intervention

All patients received cognitive behavioral therapy. Face-to-face treatment was delivered at AKiP and teletherapy was delivered via real-time, interactive videoconferencing. Teletherapy sessions were delivered in accordance with the guidelines for webcam-based telemental health of the German National Association of Statutory Health Insurance Physicians [25] using the software platform Arztkonsultation (www.arztkonsultation.de), which has been certified regarding data protection and security [26]. The therapists used a tablet or computer with webcam in the outpatient clinic. The patients and/or their parent/caregivers used their own computer with a webcam, laptop, tablet or smartphone.

### Measures

#### Corona Child Stress Scale (CCSS; [12, 42])

This questionnaire is based on the Coronavirus Health and Impact Survey (CRISIS; [33] and exists in a child (CCSS-C) and parent version (CCSS-P). For the present study, the parent version (14 items) and an analogously developed therapist version (six items) were used (see Additional file 1). Both versions contain six items regarding pandemic-related changes in family relationships, school and learning, mental health symptoms, and therapy. The parent version also assesses changes in the child’s peer relationships, daycare, leisure time, and psychopharmacotherapy, and parents’ work and family situation. Items are rated on a five-point Likert scale from − 2 to 2, with higher scores representing a higher pandemic-related burden. Internal consistency in this sample was acceptable to good (therapist rating: α = 0.78, parent rating: α = 0.87).

#### Questionnaire to assess the implementation of and satisfaction with teletherapy

This questionnaire was developed for the purpose of the study (see Additional file 1 for a full list of items and response options). It contains 15 items in the therapist version (T) and 11 in the parent version (P). At the start of both versions, respondents are asked whether teletherapy sessions have occurred since lockdown and if not, to provide reasons. Next, they are asked to indicate the number of teletherapy sessions conducted (only therapist version) and the persons involved in the teletherapy (e.g., patient, caregiver). Treatment satisfaction is assessed with seven items in the therapist version and four items in the parent version. The satisfaction items assess (a) whether the internet connection was stable, (b) the satisfaction with teletherapy, and (c) whether the respondents intend to use teletherapy after the pandemic. All satisfaction items are rated on Likert scales (0–3) (see Additional file 1). Individual scores on the satisfaction items were averaged to obtain a mean satisfaction score (MSS). In the therapist rating, there were missing data on item T6 (“patient is satisfied”, *n* = 28) and T7 (“caregiver is satisfied”, *n* = 139) because in some cases either the patient or the caregiver(s) did not participate in teletherapy. Similarly, data were missing in the parent rating for item P6 (“child is satisfied”, *n* = 24). We therefore recoded items assessing patient and caregiver satisfaction into a single item. If either the item on patient satisfaction (T6/P6) or the item on caregiver satisfaction (T7/P5) was completed, the item value of the respective item was inserted. If both items were completed, the mean value of these two items was inserted. The new item was then used to calculate the MSS.

In both the therapist and parent version, changes in treatment satisfaction and in the therapeutic relationship due the COVID-19 crisis and the changeover to teletherapy are assessed with four items rated on a five-point scale from much worse (− 2) to much better (2) (see Additional file 1). Again, data were missing if either the patient or the caregiver(s) did not participate in teletherapy (see Tables 2 and 3). The four items were thus recoded into two variables assessing (a) changes in treatment satisfaction and (b) changes in the therapeutic relationship. The mean satisfaction change score (MCS) was computed from these two items. Both scores (MSS and MCS) were used as dependent variables for further analyses. Internal consistency was acceptable to good for the therapist-rated MSS [α = 0.81 (6 items)] and the parent-rated MCS [α = 0.65 (2 items)], but unsatisfactory for the therapist-rated MCS [α = 0.59 (2 items)] and the parent-rated MSS [α = 0.57 (3 items)].

#### Basic documentation [14]

This therapist-completed scale records information about the patient (e.g., sociodemographic data, diagnosis) at the time of admission. For the present analyses, the following variables were extracted: child age, gender, living situation, socio-economic status, psychosocial functioning, and diagnosis. Psychosocial functioning rating is assessed with the Children’s Global Assessment Scale (CGAS; [40].

#### German versions of Child Behavior Checklist (CBCL/6-18R) and Youth Self Report (YSR/11-18R) [13]

These parent report (CBCL; patients ≥ 6 years) and self-report (YSR; patients ≥ 11 years) questionnaires contain 120 and 105 items, respectively, that ask about behavioral and emotional problems and physical complaints. They are rated on a three-point scale from not true (0) to very true or often true (2). Higher scores indicate greater symptom severity. Both questionnaires consist of eight problem scales, some of which are summarized into two broad-band syndrome scales assessing internalizing problems and externalizing problems. These two scales have shown good to very good internal consistency (Cronbach’s α > 0.80; [13].

#### German Symptom Checklist for Screening Behavioral and Emotional Problems (FBB-SCREEN and SBB-SCREEN; [11]

The parent-rated FBB-SCREEN (for patients ≥ 4 years) and the self-rated SBB-SCREEN (for patients ≥ 11 years) assess several symptom areas with 51 items rated from not true at all (0) to very true or often true (3). There are seven first-order scales for symptoms of ADHD, conduct disorders, anxiety, depression, developmental and elimination disorders, autism, obsessive–compulsive disorder, and tic disorders. In addition, four superordinate scales (internalizing, externalizing, contact problems, and overall symptoms) are formed, which have shown acceptable to good internal consistencies (Cronbach’s α ≥ 0.76) in community samples [11].

### Procedure

The CCSS and the study questionnaire were conducted either online [using the LimeSurvey survey tool (LimeSurvey [27])] or by paper-and-pencil. Respondents first completed the CCSS followed by the study questionnaire. Therapists received the questionnaires pseudonymized for each patient by email via the LimeSurvey tool. Between June 15 and August 25, 2020, they received several reminders to participate. All therapists completed the survey online between June 15 and August 20, 2020. Parents received the questionnaires by email or by post if no email address was available. Between July 27 and September 12, 2020, they received two reminders to participate. The participating parents completed the survey between July 27 and October 22, 2020. *N* = 162 parents (71% of *n* = 227) completed the survey online, and *n* = 65 parents (29%) completed the survey on paper.

The remaining rating scales (Basic Documentation, CBCL, YSR, FBB-SCREEN, SBB-SCREEN) are routinely collected at AKiP as part of the standard intake assessment.

### Missing values and statistical analyses

The statistical analyses were conducted using the Statistical Package for the Social Sciences, SPSS version 26 (IBM Corporation, Armonk, NY).

Two parents reported having received teletherapy but did not complete the study questionnaire. Results regarding the implementation of and satisfaction with teletherapy are, therefore, based on responses of *n* = 166 parents. There were no further missing data in the assessments with LimeSurvey (except for items that could not be completed because either patient or caregiver did not receive teletherapy, see Tables 2 and 3). Information on living situation, socio-economic status, psychosocial functioning, and ratings of mental health problems was missing in some cases (see Table 1). No missing data were replaced.

**Table 1:** Patient characteristics

Rater	Therapist- or parent-rated	Therapist-rated			Parent-rated		
		All patients	Teletherapy	No teletherapy	All patients	Teletherapy	No teletherapy
*n*	643	561	408	153	227	168	59
Age in years: mean (SD)	12.04 (4.00)	12.14 (4.03)	12.20 (4.05)	11.96 (3.96)	11.37 (3.83)	12.29 (4.01)	11.05 (3.74)
Male gender *n* (%)	362 (56.2)	306 (54.5)	219 (53.7)	87 (56.9)	142 (62.6)	107 (63.7)	34 (57.6)
Living situation *n* (%)	634 (98.6)	553 (98.6)	404 (99.0)	149 (97.4)	225 (99.1)	167 (99.4)	58 (98.3)
Single parent	108 (16.8)	97 (17.3)	62 (15.2)	35 (22.9)	28 (12.3)	19 (11.4)	9 (15.5)
Both parents	486 (75.6)	420 (74.9)	319 (782)	101 (66.0)	188 (82.8)	139 (85.3)	46 (79.3)
Other	40 (6.2)	36 (6.4)	23 (5.6)	13 (8.5)	9 (4.0)	4 (2.5)	3 (5.2)
Socio-economic status^a^: median	4	4	4	3	4	4	4
Psychosocial functioning^b^: mean (SD)	4.87 (1.10)	4.87 (1.09)	4.88 (1.09)	4.78 (1.07)	4.85 (1.08)	5.01 (1.16)	4.67 (1.22)
Diagnosis *n* (%)							
Externalizing disorder	211 (32.8)	174 (31.0)	119 (29.2)	55 (35.9)	84 (37.0)	61 (36.3)	23 (39.0)
Internalizing disorder	260 (40.4)	236 (42.1)	176 (43.1)	60 (39.2)	81 (35.7)	56 (33.3)	25 (42.4)
Externalizing and internalizing disorder	72 (11.2)	59 (10.5)	42 (10.3)	17 (11.1)	29 (12.8)	23 (13.7)	6 (10.2)
Other disorder	100 (15.6)	92 (16.4)	71 (17.4)	21 (13.7)	33 (14.5)	28 (16.7)	5 (8.5)

For living situation (*n* = 634), socio-economic status (*n* = 603), and psychosocial functioning (*n* = 639), sample size varies due to missing data

^a^Scale: 1 = unskilled workers to 6 = executives, higher-level civil servants as well as academics, liberal professions, larger entrepreneurs

^b^Scale: 0 = needs constant care to 8 = excellent or good social adaptation in all areas

The study questionnaire was descriptively analyzed on the item level. The Wilcoxon test was used to determine differences between therapist and parent ratings on single items. We calculated Pearson correlations between treatment satisfaction and children’s age, psychosocial functioning level, and psychopathology (raw scores of CBCL and YSR, scale scores of FBB-SCREEN and SBB-SCREEN). The relationship between treatment satisfaction and socio-economic status was examined using Spearman’s rank correlation.

## Results

### Participant flow and sample characteristics

**Fig. 1:**
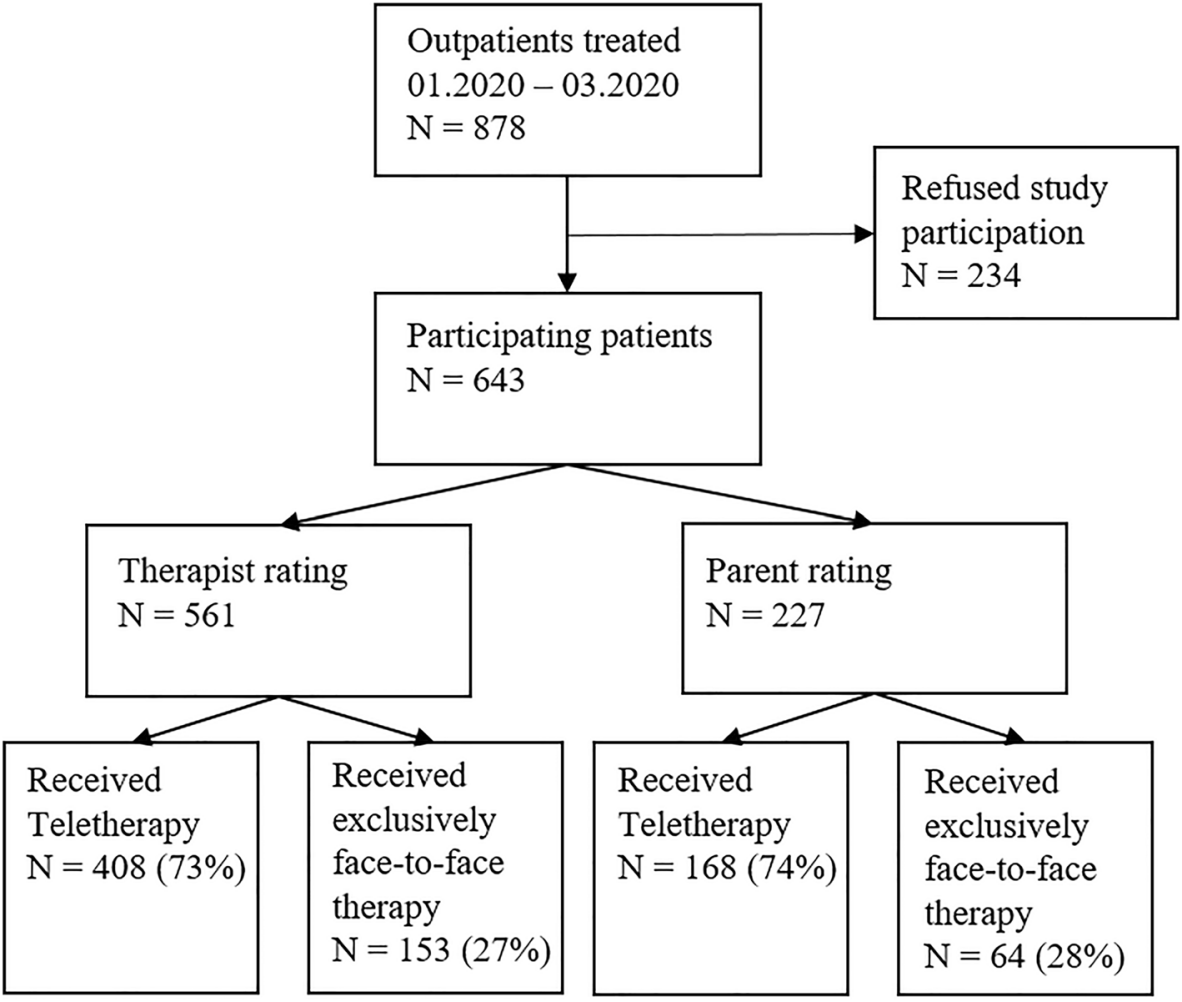
Sample recruitment

Figure 1 shows the flow of participants. *N* = 878 patients received treatment at the outpatient unit of the School of Child and Adolescent Cognitive Behavior Therapy (AKiP) at the University Hospital Cologne between January and March 2020. All patients and caregivers were informed about the study and asked to participate. A total of 643 patients (73%) provided consent and were included in the analyses. Therapist ratings were available from 215 therapists for 561 patients. Parent ratings were available for 227 patients. For 145 patients, both ratings were available.

The characteristics of the total sample with available therapist and/or parent rating (*n* = 643) and the two subsamples [parent rating (*n* = 561); therapist rating (*n* = 227)] are presented in Table 1. The patients were aged 3–20 years. There were slightly more male patients (56% males). Most of the patients lived with two biological or adoptive/foster parents. Parental socio-economic status ranged from unskilled workers to academics or larger entrepreneurs. Patients’ psychosocial functioning ranged from low to high. Approximately 33% of patients had a clinical diagnosis of an externalizing disorder (e.g., attention-deficit/hyperactivity disorder, oppositional defiant disorder, conduct disorder) at the time of admission while about 40% had an internalizing disorder (e.g., anxiety disorder, obsessive–compulsive disorder, depressive disorder).

### Implementation and acceptance of teletherapy

Over 70% of the sample (therapist rating: *n* = 408, 73%; parent rating: *n* = 168, 74%) had participated in teletherapy (see Fig. 1).

#### Reasons for not using teletherapy

In 180 patients (28% of total sample), teletherapy was not conducted. According to the therapists, the most frequently cited reasons for not switching to teletherapy were as follows: (1) the therapy had just started or was paused or terminated (32%), (2) lack of parental or patient consent (29%), (3) technical conditions were not met (19%), and (4) teletherapy was contraindicated (18%) due to the patient’s young age, severity of the disorder, or an acute crisis. Parents most frequently indicated (1) that teletherapy was not offered by the therapist (36%), (2) the therapy had just started or was paused or terminated (15%), (3) lack of parental or patient consent (10%), and (4) technical conditions were not met (6%).

#### Frequency of use of teletherapy

At the time of assessment, 190 of 215 therapists had carried out at least one teletherapy session (*M* = 6.18, *SD* = 3.63; range: 1–17 sessions per patient) within a maximum of 3 months.

Most therapists reported that if teletherapy was conducted (*n* = 408), teletherapy sessions were conducted with the patient (93.1%) and/or caregivers (mother: 63%, father: 23%, other caregiver: 4%). In 34% of cases, teletherapy was conducted only with the patient and in 5% of cases teletherapy was conducted only with the caregivers. Similarly, most parents reported that if teletherapy was conducted (*n* = 166), the sessions were conducted with the patient (84%) and/or caregivers (mother: 58%, father: 27%, other: 1%). In 41% of cases teletherapy sessions took place with the patient only and in 16% of cases teletherapy was conducted only with the caregivers.

### Satisfaction with teletherapy

#### Descriptive statistics for treatment satisfaction

Table 2 provides an overview of the descriptive statistics of therapists’ satisfaction. Overall, in 73% of cases therapists rated the internet connection and transmission quality as partially or completely sufficient. In 78%/87% of cases therapists rated the patients/caregivers to be at least partially satisfied with the teletherapy. Therapists’ own satisfaction was slightly lower, although they were at least partially satisfied in the majority of cases (73%).

**Table 2:** Satisfaction with teletherapy, item scores (therapist rating, n = 408)

Item	*N*	*M*	*SD*	*n* (%)			
				Not true (0)	Rather not true (1)	Partially true (2)	very true (3)
T5: stable internet connection	408	1.82	0.87	41 (10.0)	72 (17.6)	213 (52.2)	82 (14.6)
T6: overall satisfaction patient	380	2.02	0.86	25 (6.6)	60 (15.8)	177 (46.6)	118 (31.1)
T7: overall satisfaction caregivers	269	2.24	0.77	10 (3.7)	24 (8.9)	127 (47.2)	108 (40.1)
T8: overall satisfaction therapist	408	1.83	0.89	46 (11.3)	63 (15.4)	212 (52.0)	87 (21.3)
T9: restriction of therapeutic options (-)	408	1.99	0.79	26 (6.4)	47 (11.5)	232 (56.9)	103 (25.2)
T10: extension of therapeutic options	408	1.22	0.89	104 (25.5)	135 (33.1)	145 (35.5)	24 (5.9)

Item	*N*	*M*	*SD*	*n* (%)			
				No (0)	Yes, partly (1)	Yes, mostly (2)	Yes, exclusively (3)
T15: teletherapy in future	408	0.58	0.58	190 (46.6)	199 (48.8)	19 (4.7)	0 (0.0)
Mean satisfaction score (MSS)	408	1.42	0.62				

Item	*N*	*M*	*SD*	*n* (%)				
				Much worse (− 2)	A little worse (− 1)	Unchanged (0)	A little better (1)	Much better (2)
T11: change in patient satisfaction	380	− 0.15	0.56	6 (1.6)	74 (19.5)	272 (71.6)	28 (7.4)	0 (0.0)
T12: change in caregiver satisfaction	269	0.01	0.52	3 (1.1)	25 (9.3)	207 (77.0)	34 (12.6)	0 (0.0)
T13: change in patient-therapist relationship	380	− 0.19	0.64	8 (2.1)	92 (24.2)	246 (64.7)	31 (8.2)	3 (0.8)
T14: change in caregiver-therapist relationship	269	0.06	0.52	2 (0.7)	20 (7.4)	210 (78.1)	34 (12.6)	3 (1.1)
Mean satisfaction change score (MCS)	408	− 0.09	0.41					

*n* absolute frequencies, sample size varies due to missing data;* %* percentage frequencies*, M* mean, *SD *standard deviation

Eighty-two percent of the therapists identified therapeutic limitations due to teletherapy while 41% identified additional therapeutic options due to teletherapy. In most cases the therapists rated that the satisfaction of patients (72%) and caregivers (77%) had not changed due to the switch to teletherapy. The therapists reported more cases with reduced patient satisfaction (20%) than with improved patient satisfaction (7%). Regarding caregiver satisfaction as rated by the therapists, the rates of reduced satisfaction (10%) and improved satisfaction (13%) were similar. The therapists assessed the therapeutic relationship with the patient and with the caregiver as mostly unaffected by the change. However, there were more cases with a reduced therapist-patient relationship (26%) than with an improved therapist-patient relationship (9%). Regarding the therapist-caregiver relationship as rated by the therapists, there were somewhat fewer cases with a reduced relationship (8%) than with an improved relationship (14%). In almost half of cases (47%) therapists would not opt to continue teletherapy once face-to-face-therapy became possible again without restrictions. However, in a similar proportion of cases (53%) therapists would opt at least partially to continue to use teletherapy even if face-to-face-therapy were possible.

Table 3 depicts the descriptive statistics of the parent ratings regarding satisfaction with teletherapy. Additionally, results of the comparisons between the parent and therapist ratings (Wilcoxon test, *n* = 108) are shown. Regarding the technical conditions, parents were significantly more satisfied than the therapists. Over 82% of the parents were at least partially satisfied. In 77.0% of cases there was no change in parents’ treatment satisfaction due to the switch to teletherapy. The children’s satisfaction was rated as being slightly lower, although the majority saw no change in the child’s satisfaction due to the changeover to teletherapy (65%). The majority of parents reported that teletherapy did not influence the relationship between the therapist and themselves (87%) or their child (74%). A mixed picture emerged regarding the prospect of continuing teletherapy, with a third of parents reporting that they did not intend to use teletherapy in the future. Overall, parents reported a significantly higher intention to use teletherapy in the future than did therapists.

**Table 3:** Satisfaction with teletherapy, item scores (parent rating, n = 166)

Item	*N*	*M*	*SD*	*n* (%)				p vs. t^a^
				Not true (0)	Rather not true (1)	Partially true (2)	Very true (3)	
P4: stable internet connection	164	2.15	0.82	8 (4.9)	21 (12.8)	74 (45.1)	61 (37.2)	p > t*
P5: overall satisfaction caregivers	164	2.36	0.73	3 (1.8)	15 (9.1)	66 (40.2)	80 (48.8)	p = t
P6: overall satisfaction patient	140	2.16	0.89	11 (7.9)	13 (9.3)	59 (42.1)	57 (40.7)	p = t

Item	*N*	*M*	*SD*	*n* (%)				p vs. t^a^
				No (0)	Yes, partly (1)	Yes, mostly (2)	Yes, exclusively (3)	
P11: teletherapy in future	166	0.89	0.77	56 (33.7)	77 (46.4)	29 (17.5)	4 (2.4)	p > t*
Mean satisfaction score (MSS)	166	1.76	0.59					

Item	*N*	*M*	*SD*	*n* (%)					p vs. t^a^
				Much worse (− 2)	A little worse (− 1)	Unchanged (0)	A little better (1)	Much better (2)	
P7: change in caregiver satisfaction	165	− 0.01	0.60	2 (1.2)	19 (11.5)	127 (77.0)	12 (7.3)	5 (3.0)	p = t
P8: change in patient satisfaction	166	− 0.11	0.68	3 (1.8)	36 (21.7)	108 (65.1)	15 (9.0)	4 (2.4)	p = t
P9: change in caregiver-therapist relationship	164	0.08	0.47	1 (0.6)	5 (3.0)	142 (86.6)	12 (7.3)	4 (2.4)	p = t
P10: change in patient-therapist relationship	165	− 0.05	0.60	2 (1.2)	24 (14.5)	123 (74.5)	12 (7.3)	4 (2.4)	p = t
Mean satisfaction change score (MCS)	166	0.01	0.40						

*n* absolute frequencies, sample size varies due to missing data;* %* percentage frequencies*, M* mean, *SD* standard deviation

^a^Wilcoxon test to examine differences between the assessment of parents (p) and therapists (t), *n* = 108; *p < .05

#### Factors associated with treatment satisfaction

Correlations between treatment satisfaction and child psychopathology were low. Therapist satisfaction with teletherapy (MSS) was somewhat higher for patients with higher psychosocial functioning (*r* = 0.12, *p* = 0.02). In addition, lower therapist-rated stress due to the COVID-19 pandemic was related to higher therapist-rated treatment satisfaction (MSS: *r* = − 0.19, *p* < 0.001; MCS: *r* = − 0.20, *p* < 0.001). The number of teletherapy sessions correlated positively with therapist-rated satisfaction (MSS: *r* = 0.36, *p* < 0.001; MCS: *r* = 0.26, *p* < 0.001).

Furthermore, therapist-rated satisfaction with teletherapy (MSS) was higher in the case of children with lower parent-rated behavioral and emotional problems (CBCL Total: *r* = − 0.15, *p* < 0.01) and externalizing symptoms (CBCL Externalizing: *r* = − 0.17, *p* < 0.01; FBB-SCREEN Externalizing: *r* = − 0.11, *p* = 0.04) as well as symptoms of ADHD (FBB-SCREEN subscale ADHD: *r* = − 0.11, *p* < 0.05) and autism (FBB-SCREEN subscale autism: *r* = − 0.12, *p* = 0.03).

Therapists’ rating of change regarding satisfaction (MCS) with teletherapy was higher in the case of children with lower parent-reported externalizing symptoms (CBCL Externalizing: *r* = − 0.11, *p* = 0.04; YSR Externalizing: *r* = − 0.14, *p* = 0.04) and higher self-reported anxiety symptoms (SBB-SCREEN subscale anxiety: *r* = 0.18, *p* = 0.01).

There were no significant correlations between parent-rated treatment satisfaction (MSS, MCS) and the severity of patients’ symptoms, stress, or psychosocial functioning. The number of teletherapy sessions correlated positively with parent-rated treatment satisfaction (MSS: *r* = 0.20, *p* < 0.02).

## Discussion

The present study examined the implementation of cognitive-behavioral teletherapy. The first aim was to examine the extent to which teletherapy for children and adolescents with mental disorders was implemented and accepted within routine outpatient care under pandemic conditions. Overall, for more than 70% of all patients, teletherapy was accepted by the therapists, the patients, and the parents. Moreover, both therapists and parents who accepted teletherapy showed a robust treatment satisfaction, which did not change due to the switch to teletherapy. This is in line with data collected and published before the onset of the pandemic [9, 38], even though the switch to teletherapy within the current study was unprepared and forced. The present findings also correspond to a study conducted in a comparable German outpatient unit for cognitive-behavior therapy [3].

In the present study, parents were slightly more satisfied with teletherapy than were therapists, and saw more potential for teletherapy for the time after the COVID-19 pandemic, which is also in line with previous studies [28, 36]. This might be explained by findings that treatment barriers such as organizational aspects (e.g., travel time) may play a greater role for parents than for therapists [18, 19].

The finding that even an unprepared switch to teletherapy is accepted (and acceptance increases with increasing experience) offers a promising perspective for better-prepared teletherapy in the post-pandemic period [20, 30].

Furthermore, we examined which factors influence treatment satisfaction. We found a correlation between therapist reported treatment satisfaction and the number of teletherapy sessions. It is possible that a more frequent use of teletherapy leads to greater satisfaction, as it may take time to discover the potential advantages of teletherapy (e.g., interventions can be conducted in situations in which symptoms occur), while at the beginning, it may require effort and creativity to convert therapy methods into digital formats. Previous studies found that the therapists’ experiences play an important role in satisfaction with internet-based forms of therapy [20, 30]. It is, therefore, likely that the therapists’ prior experiences with digital technologies and their expectations about teletherapy influence their use of and their satisfaction with teletherapy. Future research is needed to explore these associations.

We also found a correlation between parent reported treatment satisfaction and the number of teletherapy sessions. This correlation may imply that satisfaction with teletherapy increases with more experience. Another possible interpretation may be that families who were satisfied with teletherapy continued with teletherapy, while families that were dissatisfied with teletherapy switched back to face-to-face treatment as soon as possible. Finally, for families who were approaching the end of treatment, a retrospective bias may have influenced parents’ reports of treatment satisfaction. To reduce this bias, we followed the recommendation to collect data on treatment satisfaction before the end of treatment [5]. Additionally, we found that more severe externalizing symptoms of the patients were associated with lower therapist-rated treatment satisfaction. A possible explanation may be that motor restlessness and inattentiveness make teletherapy difficult. A positive correlation was found between patients’ anxiety symptoms and the change of satisfaction of the therapist. In children with more severe anxiety symptoms, therapists were more likely to be more satisfied with treatment and with the therapeutic relationship after the switch to teletherapy. It is possible that teletherapy makes it easier for anxious patients to open up. Indeed, previous case reports suggested that teletherapy offers advantages for patients with social anxiety, autism, or trauma-related disorders [36].

Moreover, therapist-rated treatment satisfaction was found to be higher for children with higher psychosocial functioning and lower stress and burden due to the COVID-19 pandemic. Greater adaptability, independence, and flexibility may facilitate teletherapy with the patient.

To the best of our knowledge, the present study is one of the first to examine acceptance of and satisfaction with cognitive-behavioral routine teletherapy in a large sample of children with mental disorders. A possible limitation is that symptom ratings, psychosocial functioning, and diagnoses were recorded at the start of treatment, which may have taken place several months before the teletherapy started. A further limitation is that we did not assess patients’ self-reported treatment satisfaction due to the large number of young children in our sample (*n* = 285 patients aged ≤ 10 years). Future research is needed to investigate if and in which ways patients’ self-ratings of satisfaction with teletherapy differ from parent and therapist ratings.

Overall, although we found significant correlations between treatment satisfaction and child characteristics, the magnitude of the correlations was small. Future research is needed to explore more factors that may possibly influence satisfaction with teletherapy such as the device used for teletherapy and efforts taken to maintain the child’s focus (see for example, [8]). In addition, the time of treatment before the switch to teletherapy may influence treatment satisfaction with teletherapy and should be considered in further research.

Future research should also explore the reasons for differences in satisfaction among therapists, patients and parents using qualitative or mixed-method approaches. It has, for example, been reported that teletherapy is perceived as unsafe and less private compared to face-to-face treatment, especially in families with lower socioeconomic status [8, 23].

In principle, the general problems of satisfaction surveys (e.g., patients usually report high satisfaction with the treatment they receive) should also be taken into account [34], and may have led to an overestimation of satisfaction. However, the questionnaire included items assessing teletherapy in comparison to previous direct therapy, and here too, there was no deterioration.

The current study included only children and adolescents who were already in psychotherapy and knew their therapists beforehand, which may imply that a therapeutic relationship had already been established. In a qualitative study, all patients named the therapeutic relationship as an important factor for the acceptance of teletherapy [4, 37]. Therefore, it would be interesting to examine the therapeutic relationship in patients who have not previously met their therapists in face-to-face sessions.

## Conclusions

The present study is among the first of its kind to show the feasibility and acceptance of teletherapy, assessed by therapists and parents, during the switch due to the COVID-19 pandemic in such a large sample. The findings support initial indications that the challenges of this switch are manageable and that for most patients, a continuation of psychotherapeutic care is possible in times of crisis [6, 41].

Teletherapy has great potential for the future beyond the COVID-19 pandemic, but the pandemic has hugely fostered its implementation and examination. Despite the immense challenges of the pandemic, teletherapy as a stand-alone treatment or add-on to face-to-face psychotherapy (blended therapy), as preferred by most therapists and parents, should be seen as an opportunity to expand and improve therapeutic care in the long term.

## Supplementary Information

**Additional file 1.** Corona Child Stress Scale and Questionnaire to assess the implementation of and satisfaction with teletherapy.

## Data Availability

The datasets generated and analyzed during this study are not publicly available. But are available from the corresponding author on reasonable request.

## Abbreviations

AKiP: School for Child and Adolescent Cognitive Behavior Therapy at the University Hospital Cologne, Germany
CBCL: Child Behavior Checklist
CCSS: Corona Child Stress Scale
FBB-SCREEN: German Symptom Checklist for Screening Behavioral and Emotional Problems—parent rating
KBV: Kassenärztlichen Bundesvereinigung (Engl: German National Association of Statutory Health Insurance Physicians)
SBB-SCREEN: German Symptom Checklist for Screening Behavioral and Emotional Problems—self rating
YSR: Youth Self Report
