# Supplementary material for: Cognitive-behavioral teletherapy for children and adolescents with mental disorders and their families during the COVID-19 pandemic: a survey on acceptance and satisfaction
Source: Child Adolesc Psychiatry Ment Health. 2022 Jul 28;16:61. doi: 10.1186/s13034-022-00494-7 (PMC9330974; doi:10.1186/s13034-022-00494-7)
Supplement: Supplementary file 1 — Additional file 1. Corona Child Stress Scale and Questionnaire to assess the implementation of and satisfaction with teletherapy. [file 13034_2022_494_MOESM1_ESM.docx]

Electronic Supplement (ESM)

*Corona Child Stress Scale (CCSS)*

*Parent version*

| 1 | Have the relationships between your child and the other family members changed due to COVID-19? |
| --- | --- |
| 2 | Have the relationships between your child and his / her friends changed due to COVID-19? |
| 3 | Has your child’s burden regarding school and learning changed due to COVID-19? |
| 4 | Has your child’s daycare (apart from school) changed due to COVID-19? |
| 5 | Have the leisure options for your child changed due to COVID-19? |
| 6 | Is your child more irritable, moody, or restless due to the COVID-19 crisis? |
| 7 | Is your child more anxious, insecure, or sad due to the COVID-19 crisis? |
| 8 | Have the individual problems of your child, which were the main motive for therapy, changed during the COVID-19 crisis? |
| 9 | Has the extent to which you or your child implements strategies taught in therapy changed due to the COVID-19 crisis? |
| 10 | If child receives psychopharmacotherapy: Has the psychopharmacotherapy of your child changed due to the COVID-19 crisis? |
| 11 | Has your work situation changed due to the COVID-19 crisis? |
| 12 | Has your family situation changed due to the COVID-19 crisis? |
| 13 | How would you describe the changes due to the COVID-19 crisis for your child in general? |
| 14 | How would you describe the changes due to the COVID-19 crisis for yourself in general? |

*Therapist version*

| 1 | Have the relationships between the patient and the other family members changed due to COVID-19? |
| --- | --- |
| 2 | Has the patient’s burden regarding school and learning changed due to COVID-19? |
| 3 | Is the patient more irritable, moody, or restless due to the COVID-19 crisis? |
| 4 | Is the patient more anxious, insecure, or sad due to the COVID-19 crisis? |
| 5 | Have the individual problems of the patient, which were the main motive for therapy, changed during the COVID-19 crisis? |
| 6 | Has the extent to which the patient or his / her parents implement strategies taught in therapy changed due to the COVID-19 crisis? |

*Questionnaire to assess the implementation of and satisfaction with teletherapy*

*Parent version*

| Implementation items | | |
| --- | --- | --- |
| P1 | Have you received teletherapy sessions? | Yes / No |
| P2 | If teletherapy has not been conducted: What were the reasons? | - Treatment had ended - Technical conditions were not met (e.g., no internet access) - Therapist did not offer teletherapy - I did not agree to teletherapy - Other: ____________ |
| P3 | Who participated in teletherapy sessions? | - mother - father - patient - other caregiver |
| Satisfaction items | | |
| P4 | The connection was stable (no image / sound interferences or connection failures). | not true (0) to very true (3) |
| P5 | I am satisfied with the teletherapy. | not true (0) to very true (3) |
| P6 | My child is satisfied with the teletherapy. | not true (0) to very true (3) |
| P11 | I intend to use teletherapy in the future, even if sessions can be held face to face. | No (0)  Yes, partly (1)  Yes, mostly (2)  Yes, exclusively (3) |
| Satisfaction change items | | |
| P7 | Has your satisfaction with the treatment changed due to the COVID-19 crisis and the changeover to teletherapy? | Much worse (-2)  A little worse (-1)  Unchanged (0)  A little better (1)  Much better (2) |
| P8 | Has your child’s satisfaction with the treatment changed due to the COVID-19 crisis and the changeover to teletherapy? | Much worse (-2)  A little worse (-1)  Unchanged (0)  A little better (1)  Much better (2) |
| P9 | Has the therapeutic relationship between you and the therapist changed due to the COVID-19 crisis and the changeover to teletherapy? | Much worse (-2)  A little worse (-1)  Unchanged (0)  A little better (1)  Much better (2) |
| P10 | Has the therapeutic relationship between your child and the therapist changed due to the COVID-19 crisis and the changeover to teletherapy? | Much worse (-2)  A little worse (-1)  Unchanged (0)  A little better (1)  Much better (2) |

*Therapist version*

| Implementation items | | |
| --- | --- | --- |
| T1 | Have you conducted teletherapy sessions with the patient or his/her caregivers? | Yes / No |
| T2 | If teletherapy has not been conducted: What were the reasons? | - Treatment had ended - Technical conditions were not met (e.g., no internet access) - Parents/caregivers did not agree to teletherapy because: _____________________ - Teletherapy was not indicated because: _____________________ - I declined teletherapy because: _____________________ - Other: _____________________ |
| T3 | How many sessions have you conducted via videoconferencing (teletherapy?) |  |
| T4 | Who participated in teletherapy sessions? | - mother - father - patient - other caregiver |
| Satisfaction items | | |
| T5 | The connection was stable (no image / sound interferences or connection failures). | not true (0) to very true (3) |
| T6 | The patient is satisfied with the teletherapy. | not true (0) to very true (3) |
| T7 | The caregivers are satisfied with the teletherapy. | not true (0) to very true (3) |
| T8 | I am satisfied with the teletherapy. | not true (0) to very true (3) |
| T9 | Teletherapy restricts my therapeutic options for this patient. | not true (0) to very true (3) (*recoded*) |
| T10 | Teletherapy extends my therapeutic options for this patient. | not true (0) to very true (3) |
| T15 | I intend to use teletherapy with this patient in the future, even if sessions can be held face to face. | No (0)  Yes, partly (1)  Yes, mostly (2)  Yes, exclusively (3) |
| Satisfaction change items | | |
| T11 | Has the patient’s satisfaction with the treatment changed due to the COVID-19 crisis and the changeover to teletherapy? | Much worse (-2)  A little worse (-1)  Unchanged (0)  A little better (1)  Much better (2) |
| T12 | Has the caregivers’ satisfaction with the treatment changed due to the COVID-19 crisis and the changeover to teletherapy? | Much worse (-2)  A little worse (-1)  Unchanged (0)  A little better (1)  Much better (2) |
| T13 | Has the therapeutic relationship with the patient changed due to the COVID-19 crisis and the changeover to teletherapy? | Much worse (-2)  A little worse (-1)  Unchanged (0)  A little better (1)  Much better (2) |
| T14 | Has the therapeutic relationship with the caregivers changed due to the COVID-19 crisis and the changeover to teletherapy? | Much worse (-2)  A little worse (-1)  Unchanged (0)  A little better (1)  Much better (2) |
